# Long non-coding RNA MEG3 serves as a ceRNA for microRNA-145 to induce apoptosis of AC16 cardiomyocytes under high glucose condition

**DOI:** 10.1042/BSR20190444

**Published:** 2019-06-07

**Authors:** Yiwei Chen, Zhifang Zhang, Diqi Zhu, Wenchuo Zhao, Fen Li

**Affiliations:** Department of Cardiology, Shanghai Children’s Medical Center Affiliated to Shanghai Jiaotong University School of Medicine, Shanghai 200127, P.R. China

**Keywords:** diabetic cardiomyopathy, long non-coding RNA MEG3, microRNA-145, PDCD4

## Abstract

Diabetic cardiomyopathy (DCM) is one of the most serious complications of diabetes, but its pathogenesis remains largely unclear. In the present study, we aimed to explore the potential role of long non-coding RNA (lncRNA) maternally expressed gene 3 (MEG3) and to investigate the underlying mechanisms in human AC16 cardiomyocytes under high glucose (HG) condition. The results demonstrated that MEG3 was overexpressed in HG-treated AC16 cells, and MEG3 knockdown suppressed the HG-induced apoptosis in AC16 cells. Mechanistically, MEG3 directly binds to miR-145 in AC16 cells, thereby up-regulating the expression of PDCD4. Rescue experiments showed that the role of MEG3 in HG-treated AC16 cells was partly dependent on its suppression on miR-145. In summary, our findings suggested that the role of MEG3 in HG-treated human cardiomyocytes is to serve as a competing endogenous RNA (ceRNA), which negatively regulates miR-145. These findings may provide a valuable and promising therapeutic target for the treatment of DCM in the future.

## Introduction

Diabetes mellitus (DM), a metabolic disorder characterized by hyperglycemia, is an emerging global health problem. Diabetic cardiomyopathy (DCM), a common cardiovascular complication occurring in patients with DM, is featured by early impairments in diastolic function, accompanied by the development of cardiac hypertrophy, myocardial fibrosis, and cardiomyocyte apoptosis [1]. DCM is also verified as a leading cause of heart failure and mortality in diabetic individuals [2]. Despite advances in molecular etiologies, at present, the molecular mechanisms underlying DCM remain largely unclear.

Long non-coding RNAs (lncRNAs) are a group of transcribed RNA molecules with more than 200 nucleotides in length and little or no protein-coding potential. At present, lncRNAs have attracted widespread attention for their regulatory role in a wide range of biological processes and human diseases [3]. Maternally expressed gene 3 (MEG3), a typical lncRNA located in the imprinted DLK1-MEG3 locus on human chromosome 14q32.3 region, often serves as a tumor suppressor in many cancers [4]. Also, Wu et al. [5] found a remarkable up-regulation of MEG3 in mouse injured heart after myocardial infarction, and Gong et al. [6] reported that knockdown of MEG3 decreased hypoxia-induced injury in rat cardiomyocyte-derived H9c2 cells. In the present study, through a series of *in vitro* experiments, we aimed to investigate the potential regulatory role of MEG3 in human AC16 cardiomyocytes under high glucose (HG) condition.

## Materials and methods

### Cell culture and treatments

Human adult ventricular cardiomyocyte cell line AC16 was obtained from American Type Culture Collection (ATCC; Manassas, VA, U.S.A.). Cells were cultured in Dulbecco’s modified Eagle’s medium (DMEM; Invitrogen, Carlsbad, CA, U.S.A.) containing 10% fetal bovine serum (FBS; HyClone, Logan, UT, U.S.A.) and 1% penicillin/streptomycin in a humidified incubator with 5% CO_2_ at 37°C. In the following experiments, AC16 cells were exposed to 30 mmol/l glucose (HG) or 5.5 mmol/l glucose (normal glucose, NG) for 6, 12, 24 and 48 h.

Three small interfering RNAs (siRNAs) specifically targeting MEG3 were designed, and their sequences were listed as follows: si-MEG3-1: 5′-GAAGAGGCUGCAGACGUUA-3′; si-MEG3-2: 5′-GCUGCAGACGUUAAUGAGG-3′; si-MEG3-3: 5′-UGCAGACGUUAAUGAGGUU-3′. miR-145 mimics, miR-145 inhibitor and negative control oligonucleotides (NC) were designed and synthesized by GenePharma Co., Ltd. (Shanghai, China). Cells were cultured overnight to reach 80% confluence, and transfection was performed using Lipofectamine 2000 (Invitrogen). The transfection efficacy was determined by RT-qPCR analysis. Twenty-four hours after transfection, the cells were subjected to HG treatment.

### RNA extraction and RT-qPCR analysis

Total RNA was isolated from cells using TRIzol Reagent (Invitrogen). Cytoplasmic and nuclear RNA were separated and extracted using NE-PER Nuclear and Cytoplasmic Extraction Reagent (Thermo Fisher Scientific, Inc., Waltham, MA, U.S.A.). RNA was then reverse transcribed to cDNA using the PrimeScript RT reagent Kit (TaKaRa, Dalian, China). qPCR reactions were then carried out on a 7500HT Real-Time PCR System (Applied Biosystems, Foster City, CA, U.S.A.) using SYBR Green PCR Master Mix (Applied Biosystems). The relative expression of genes was calculated using the 2^−ΔΔ*C*^_t_ method [7], and GAPDH or U6 was used as an internal control for normalization. The primer sequences were listed in Table 1.

**Table 1:** The sequences of primers

Gene name	Primer sequences
*miR-145*-RT	5′-GTCGTATCCAGTGCAGGGTCCGAGGTATTCGCACTGGATACGACAGGGAT-3′
*U6*-RT	5′-GTCGTATCCAGTGCAGGGTCCGAGGTATTCGCACTGGATACGACAAAATA-3′
*miR-145* Forward primer	5′-GTCCAGTTTTCCCAGGA-3′
*miR-145* Reverse primer	5′-GTGCAGGGTCCGAGGT-3′
*U6* Forward primer	5′-CTCGCTTCGGCAGCACATATACT-3′
*U6* Reverse primer	5′-ACGCTTCACGAATTTGCGTGTC-3′
*MEG3* Forward primer	5′-CTGCCCATCTACACCTCACG-3′
*MEG3* Reverse primer	5′-CTCTCCGCCGTCTGCGCTAGGGGCT-3′
*GAPDH* Forward primer	5′-GACTCATGACCACAGTCCATGC-3′
*GAPDH* Reverse primer	5′-AGAGGCAGGGATGATGTTCTG-3′

### MTT assay

Cell viability was assessed by 3-(4,5-dimethylthiazol-2-yl)-2,5-diphenyltetrazolium bromide (MTT) colorimetric assay [8]. Cells were plated in 96-well plates (5 × 10^3^ cells/well). After the aforementioned treatments, the medium were replaced by fresh medium containing 20 μl MTT (5 mg/ml; Sigma–Aldrich, St. Louis, MO, U.S.A.). After 4 h of incubation, the formazan crystal was dissolved in 150 μl DMSO (Sigma–Aldrich), and the absorbance of each well at 490 nm was then recorded using a microplate reader (Molecular Devices, Sunnyvale, CA, U.S.A.).

### Cell apoptosis assay

Cell apoptosis was measured using the Annexin V-FITC/propidium iodide (PI) double staining kit (Beyotime, Shanghai, China). After the aforementioned treatments, cells were harvested, washed twice with ice-cold PBS, resuspended in 100 μl binding buffer, and then double stained with 10 μl Annexin V-FITC and 5 μl PI for 10 min at 4°C in the dark. Cells were then subjected to flow cytometry (FACSCalibur; Becton Dickinson, San Jose, CA, U.S.A.).

### Western blot analysis

Cells were collected and lysed using RIPA protein extraction reagent (Beyotime). Equal amounts of total protein were separated by SDS/polyacrylamide gel electrophoresis and transferred on to PVDF membranes (Millipore, Billerica, MA, U.S.A.). The membranes were then blocked and probed with specific primary antibodies at 4°C overnight. Subsequently, the membranes were incubated with HRP–conjugated secondary antibody at room temperature for 1 h. The protein bands were visualized using an enhanced chemiluminescence kit (Santa Cruz Biotechnology, Dallas, TX, U.S.A.). GAPDH was used as the internal loading control.

### Dual-luciferase reporter assay

The fragment of MEG3 or the 3′-UTR of PDCD4 containing the predicted miR-145 binding sites was amplified by PCR and respectively cloned into the pmirGLO vector (Promega, Madison, WI, U.S.A.). The binding sites were mutated using the QuickChange® Site-Directed Mutagenesis Kit (Stratagene, La Jolla, CA, U.S.A.). Cells were seeded into 24-well plates and then co-transfected with the reporter vectors and miR-145 mimics or NC using Lipofectamine 2000. Forty-eight hours after transfection, cells were harvested, and the firefly and *Renilla* luciferase activities were determined using the Dual-luciferase reporter system (Promega).

### Statistical analysis

All statistical analyses were performed using GraphPad Prism 6.0 software (GraphPad Software Inc., San Diego, CA, U.S.A.). The experimental data are expressed as mean ± standard deviation (SD), and the differences between groups were analyzed using Student’s *t* test or one-way analysis of variance (ANOVA). All *P*-values were two-sided and *P*<0.05 was considered to indicate a statistically significant difference.

## Results

### MEG3 is overexpressed in HG-treated AC16 cells

**Figure 1:**
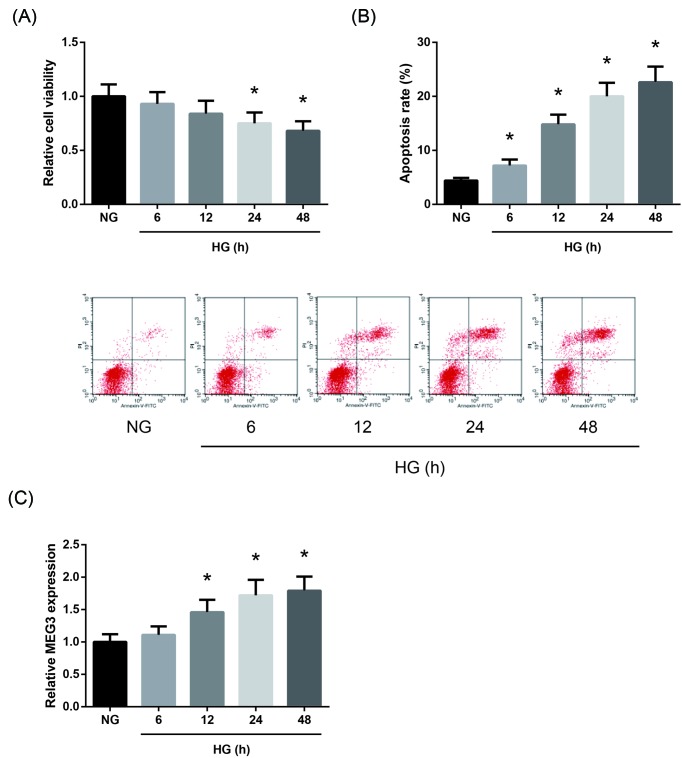
MEG3 is overexpressed in HG-treated AC16 cells (**A**) Viability of NG-treated and HG-treated AC16 cells, assessed by MTT assay. (**B**) Apoptosis of NG-treated and HG-treated AC16 cells, measured by flow cytometry. (**C**) Expression levels of MEG3 in NG-treated and HG-treated AC16 cells, detected by RT-qPCR analysis. The data are expressed as mean ± SD. *P<0.05 versus NG-treated cells.

To investigate the potential role of MEG3 in DCM, we established an *in vitro* model of DCM by using AC16 cells subjected to HG treatment. As exhibited in Figure 1A, AC16 cells following HG treatment showed decreased cell viability at 12, 24, and 48 h compared with NG-treated cells. We also observed that HG treatment increased the apoptosis rate of AC16 cells in a time-dependent manner (Figure 1B). In addition, RT-qPCR analysis revealed that the expression levels of MEG3 were time-dependently increased in HG-treated AC16 cells (Figure 1C).

### MEG3 knockdown represses the HG-induced apoptosis in AC16 cells

**Figure 2:**
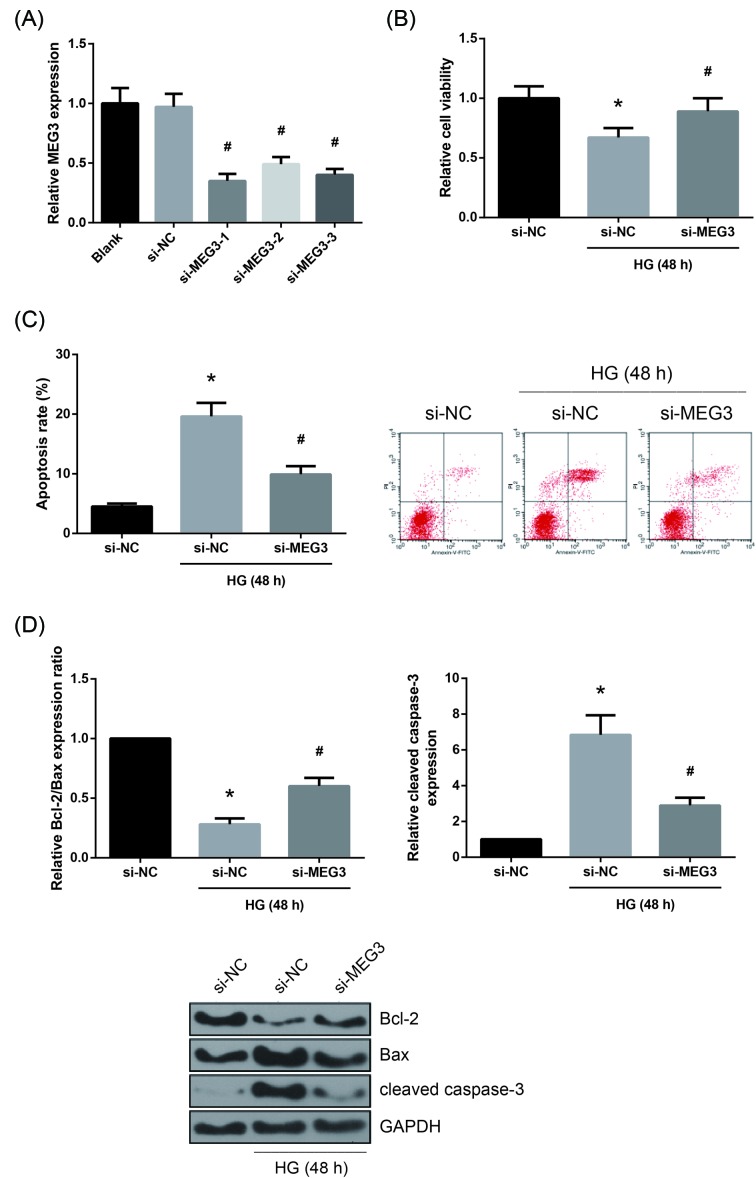
MEG3 knockdown represses the HG-induced apoptosis in AC16 cells (**A**) Expression levels of MEG3 in HG-treated AC16 cells after transfection, detected by RT-qPCR analysis. (**B**) Viability of HG-treated AC16 cells after transfection, assessed by MTT assay. (**C**) Apoptosis of HG-treated AC16 cells after transfection, measured by flow cytometry. (**D**) Expression levels of Bcl-2, Bax and cleaved caspase-3 proteins in HG-treated AC16 cells after transfection, detected by Western blot analysis. The data are expressed as mean ± SD. *P<0.05 versus NG-treated cells, ^#^*P*<0.05 versus si-NC-transfected cells.

Next, we explored the functional involvement of MEG3 in HG-treated AC16 cells by performing loss-of-function experiments. We designed three siRNAs to knockdown MEG3 level, and as shown in Figure 2A, si-MEG3-1 showed the highest knockdown efficacy in HG-treated AC16 cells, and therefore it was selected for further use. Knockdown of MEG3 blocked the inhibitory role of HG on the viability of AC16 cells, as indicated by MTT assay (Figure 2B). We also confirmed that MEG3 knockdown could suppress the HG-induced apoptosis in AC16 cells (Figure 2C). Besides, Western blot analysis pointed out that the Bcl-2/Bax expression ratio was decreased, whereas the cleaved caspase-3 expression was increased in AC16 cells following 48 h of HG treatment, but these effects were obviously restored by MEG3 knockdown (Figure 2D).

### miR-145 directly binds to MEG3 in AC16 cells

**Figure 3:**
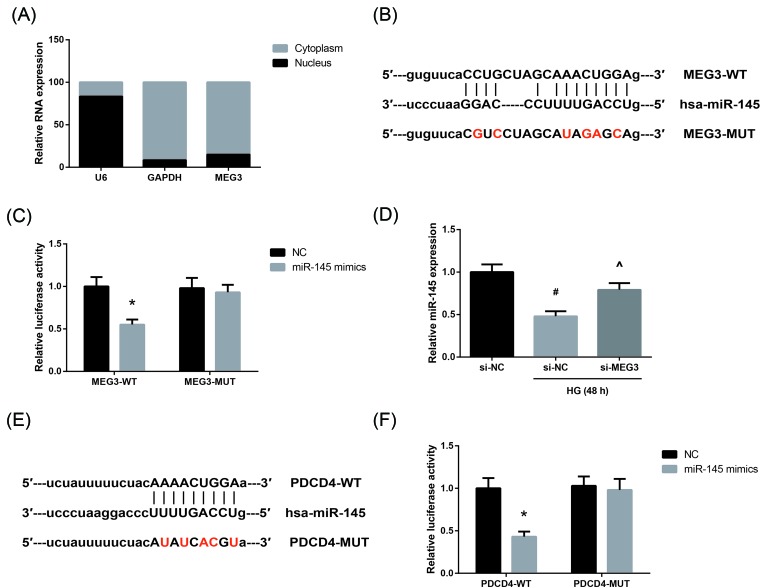
miR-145 directly binds to MEG3 in AC16 cells (**A**) Subcellular locations of MEG3 in AC16 cells, detected by RT-qPCR analysis. (**B**) Diagrammatic sketch of the binding sites for miR-145 in MEG3. (**C**) The relative luciferase activities in AC16 cells co-transfected with MEG3-WT or MEG3-MUT reporter vectors and miR-145 mimics or negative control. (**D**) Expression levels of miR-145 in HG-treated AC16 cells after transfection, detected by RT-qPCR analysis. (**E**) The miR-145-binding sites in the 3′-UTR of PDCD4 are shown. (**F**) The relative luciferase activities in AC16 cells co-transfected with PDCD4-WT or PDCD4-MUT reporter vectors and miR-145 mimics or negative control. The data are expressed as mean ± SD. *P<0.05 versus negative control-transfected cells, ^#^*P*<0.05 versus NG-treated cells, ^∧^*P*<0.05 versus si-NC-transfected cells.

LncRNAs could act as a competing endogenous RNA (ceRNA) to interact with miRNAs. Subcellular fractionation and RT-qPCR analysis showed that MEG3 was predominately localized in the cytoplasm of AC16 cells (Figure 3A), providing prerequisite for reciprocal interaction between MEG3 and miRNAs. Through the Starbase database (http://starbase.sysu.edu.cn/index.php), miR-145 was found to potentially bind to MEG3 with the putative binding sites presented in Figure 3B. To verify the prediction, we carried out dual-luciferase reporter assay, and the results demonstrated that the relative luciferase activity was significantly suppressed in AC16 cells co-transfected with MEG3-WT vector and miR-145 mimics (Figure 3C), but this repressive effect was abrogated by mutation of the binding sites. Moreover, we also noticed that the reduction of miR-145 expression in HG-treated AC16 cells was blocked by transfection with si-MEG3 (Figure 3D).

In addition, through the TargetScan database (http://www.targetscan.org), we also found that miR-145 potentially targets the 3′-UTR of PDCD4 (Figure 3E), and dual-luciferase reporter assay showed that co-transfection with PDCD4-WT vector and miR-145 mimics notably reduced the relative luciferase activity in AC16 cells (Figure 3F).

### miR-145 inhibition blocks the role of MEG3 knockdown in HG-treated AC16 cells

**Figure 4:**
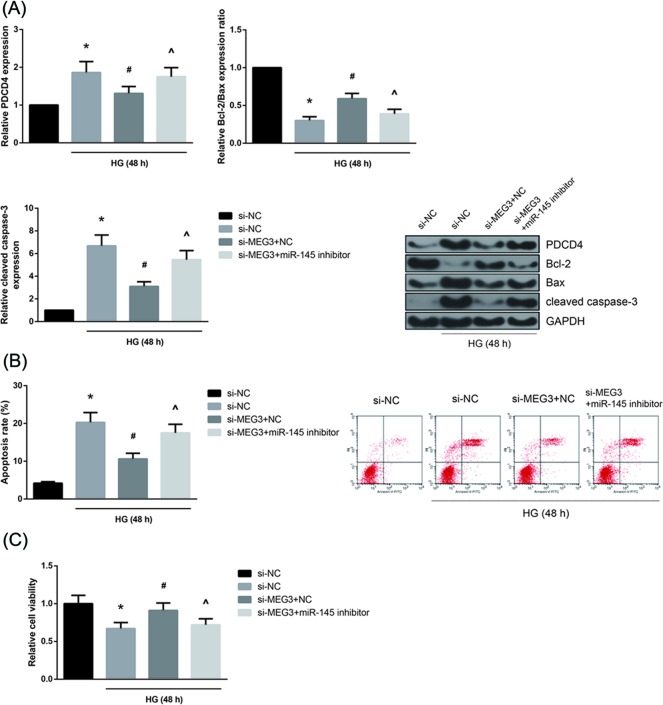
miR-145 inhibition blocks the role of MEG3 knockdown in HG-treated AC16 cells (**A**) Expression levels of PDCD4, Bcl-2, Bax and cleaved caspase-3 proteins in HG-treated AC16 cells after co-transfection, detected by Western blot analysis. (**B**) Apoptosis of HG-treated AC16 cells after co-transfection, measured by flow cytometry. (**C**) Viability of HG-treated AC16 cells after co-transfection, assessed by MTT assay. The data are expressed as mean ± SD. *P<0.05 versus NG-treated cells, ^#^*P*<0.05 versus si-NC-transfected cells, ^∧^*P*<0.05 versus si-NC+negative control-transfected cells.

To investigate whether miR-145 can functionally reverse the role of MEG3 in HG-treated AC16 cells, rescue experiments were then performed. As shown in Figure 4A, MEG3 knockdown repressed PDCD4 protein expression in HG-treated AC16 cells, and this effect was blocked by co-transfection with miR-145 inhibitor. In addition, the increased Bcl-2/Bax expression ratio and the decreased cleaved caspase-3 expression were also restored by miR-145 inhibition. Furthermore, as expected, miR-145 inhibition increased the apoptosis of HG-treated AC16 cells with MEG3 knockdown (Figure 4B). Also, MTT assay indicated that the beneficial effect of MEG3 knockdown on the viability of HG-treated AC16 cells was counteracted by miR-145 inhibition (Figure 4C).

## Discussion

The pathophysiological mechanisms underlying DCM are complex and multifactorial. In recent years, some literatures have focused on the regulatory role of lncRNAs in DCM. For example, lncRNA HOTAIR expression was significantly decreased in diabetic mice hearts [9], whereas lncRNA MIAT knockdown could reduce cardiomyocyte apoptosis and improve left ventricular function in diabetic rats [10]. The characterization of DCM-related lncRNAs is therefore critically needed to identify therapeutic targets for this serious pathological condition.

Herein, human AC16 cells were exposed to high concentration of glucose to mimic DCM *in vitro*. Apoptosis is a form of programmed cell death, and cardiomyocyte apoptosis is a critical pathological change involved in DCM [11,12]. Thus, how to reduce HG-induced cardiomyocyte apoptosis is important for the clinical therapy of DCM. In the present study, we observed a significant up-regulation of MEG3 in HG-treated AC16 cells, and the HG-induced cellular toxicity in AC16 cells was blocked by MEG3 knockdown, as evidenced by the decreased apoptosis and increased viability. The ratio of Bcl-2 to Bax controls caspase activation and determines the cell fate [13], and our study also showed that MEG3 knockdown protected AC16 cells from HG-induced apoptosis by regulating anti (pro)-apoptotic proteins.

miRNAs are another type of non-coding RNAs, which exert their functions by negative regulation of their target genes [14]. Mounting evidence has strongly implied that lncRNAs could serve as ceRNAs of miRNAs to relieve the expression and function of downstream mRNAs [15,16]. In this study, the results of bioinformatics analysis predicted that miR-145 might contain the binding sites with MEG3, and this prediction was further proved by the experimental validation. Consistent with the findings of Wang et al. [17], we also observed the reduction of miR-145 expression in HG-treated AC16 cells, and this reduction was overturned by MEG3 knockdown.

Moreover, in the present study, PDCD4 was identified as a direct target of miR-145 in AC16 cells. PDCD4 is a critical mediator of cell apoptosis. It has been previously reported that miR-145 could protect against rat myocardial infarction by targeting PDCD4 [18]. Through rescue experiments, we further validated that MEG3 might serve as a ceRNA to inhibit miR-145 expression, thereby reducing the inhibition of miR-145 on PDCD4 expression in HG-treated AC16 cells.

In conclusion, our study might be the first to demonstrate that MEG3 knockdown protected human cardiomyocytes from HG-induced apoptosis partly by regulating miR-145/PDCD4 axis. Hence, targeting MEG3 seems as a novel therapeutic strategy to protect against DCM development.

## Abbreviations

ceRNA: competing endogenous RNA
DCM: diabetic cardiomyopathy
DM: diabetes mellitus
HG: high glucose
lncRNA: long non-coding RNA
MEG3: maternally expressed gene 3
NG: normal glucose
siRNA: small interfering RNA
